# Complexities of X chromosome inactivation status in female human induced pluripotent stem cells—a brief review and scientific update for autism research

**DOI:** 10.1186/s11689-016-9155-8

**Published:** 2016-06-09

**Authors:** Mary G. Dandulakis, Kesavan Meganathan, Kristen L. Kroll, Azad Bonni, John N. Constantino

**Affiliations:** School of Medicine, Washington University in St. Louis, St. Louis, USA; Department of Developmental Biology, Washington University in St. Louis, Campus Box 8103, 660 S. Euclid Ave., St. Louis, MO 63110-1093 USA; Department of Neuroscience, Washington University in St. Louis, Campus Box 8108, 660 S. Euclid Ave., St. Louis, MO 63110-1093 USA; Department of Psychiatry, Washington University in St. Louis, Campus Box 8134, 660 S. Euclid Avenue, St. Louis, MO 63110 USA

**Keywords:** ASD, Autism, iPSC, X-inactivation, X-reactivation, Developmental disorders, X chromosome, X-linked ASD, “Female protective effect”

## Abstract

Induced pluripotent stem cells (iPSCs) allow researchers to make customized patient-derived cell lines by reprogramming noninvasively retrieved somatic cells. These cell lines have the potential to faithfully represent an individual’s genetic background; therefore, in the absence of available human brain tissue from a living patient, these models have a significant advantage relative to other models of neurodevelopmental disease. When using human induced pluripotent stem cells (hiPSCs) to model X-linked developmental disorders or inherited conditions that undergo sex-specific modulation of penetrance (e.g., autism spectrum disorders), there are significant complexities in the course and status of X chromosome inactivation (XCI) that are crucial to consider in establishing the validity of cellular models. There are major gaps and inconsistencies in the existing literature regarding XCI status during the derivation and maintenance of hiPSCs and their differentiation into neurons. Here, we briefly describe the importance of the problem, review the findings and inconsistencies of the existing literature, delineate options for specifying XCI status in clonal populations, and develop recommendations for future studies.

## Background

Approximately one third of autism spectrum disorder (ASD) patients have an identifiable pathogenic genetic variant, and as sequencing becomes more widespread, this percentage increases [1]. Although mouse models of ASD-associated mutations have successfully reconstituted some ASD phenotypes, they are limited by constraints in recapitulating complex human behaviors, by the tendency for replicable phenotypes to be most robust in homozygous knockouts, which exaggerate deficiency states, and by the inability to recapitulate background genetic factors that influence autistic syndromes in individual human patients [2]. Induced pluripotent stem cells, which are derived from reprogrammed somatic cells, offer researchers studying autism a reliable method to obtain genetically identical neurons from noninvasively retrieved patient samples. A small number of recent papers have begun to explore cellular phenotypes of autism observed in human induced pluripotent stem cell (hiPSC)-derived neurons [2–6]. HiPSC models not only offer researchers a method to elucidate the molecular biology of ASD, but they also provide a means of identifying and testing drug therapies.

An important aspect of any cellular model of disease involves the issue of whether the cells faithfully reflect the normative process of X chromosome inactivation. Although only a minority of ASD susceptibility loci reside on the X chromosome, X-linked mutations are well-known causes of related developmental disorders. Perhaps even more importantly, there is growing evidence that supports the hypothesis that there is a “female protective effect” in which the pronounced sex ratio universally observed in ASD is resolvable to the interaction between female sex and inherited autism liabilities, which are predominantly polygenic and autosomal in nature [7–9]. A proposed mechanism for this phenomenon is that genetic loci expressed on the noninactivated X chromosome modulate the phenotypic expression of autosomal loci conferring risk for ASD, which would make faithful recapitulation of X-inactivation critical to the development of valid cellular models of the condition [7]. Here, we review what is known about this important detail of hiPSC-derived cellular models, based upon the current literature.

Random X chromosome inactivation in female humans occurs early during the developmental process, resulting in the equalization of X chromosome-linked gene dosages across sexes. There are four steps associated with X chromosome inactivation (XCI), referred to as counting, choosing, initiation, and maintenance. All but maintenance are orchestrated by the X-inactivation center (Xic), which contains the noncoding RNA-encoding X-inactive specific transcript (Xist) locus. Xist RNA surrounds the X chromosome selected to be inactivated and is necessary for the initiation of its silencing. Although the exact mechanism involved in counting remains elusive, there is evidence to suggest that the cell utilizes the ratio between X chromosome number and haploid autosomal chromosome number in order to ensure that only one X chromosome is active [10]. This process is so precise that only one X chromosome is active even in instances where individuals have more than two X chromosomes [11]. Xist expression is crucial for initiation of silencing in developing embryos. However, once cells have differentiated, Xist is unable to induce inactivation [12]. Xist forms a “cloud” around the inactivated X chromosome by binding areas along the length of the chromosome, which are located near regions where Xist is actively transcribed [13]. This results in the loss of RNA polymerase II, initiation factors, and other components necessary for transcription in the areas covered by Xist [12]. However, there are regions on the inactivated X chromosome that sustain active transcription and “escape” inactivation by looping out of the chromosomal territory covered by Xist [13].

Early in development, when Xist plays the major role in inactivation, XCI is reversible. Once the initiation phase transitions to the maintenance phase, however, the inactivation status is sustained [12]. This progression is characterized by alteration in DNA methylation and histone modification. The Polycomb group (PcG) complex, a protein complex that mediates repressive histone 3 lysine 27 trimethylation (H3K27me3 ) to silence transcription, drives some of these epigenetic changes [12].

Generally, X-inactivation is a random process that results in equal expression of both X chromosomes in tissue [10]. However, under certain circumstances, one X chromosome is preferentially inactivated, which may occur when one of the X chromosomes actively expresses a severely detrimental allele [14]; such “skewing” of XCI, however, has not been consistently observed in samples of patients with ASD [15]. There are two methods that result in nonrandom expression of an X chromosome. The first method arises when the cells preferentially inactivate one X chromosome over the other. The second method occurs when cells expressing a particular X chromosome are selected against [14].

The extent to which these processes are recapitulated in the development of human-induced pluripotent stem cell-derived neuronal cell models is currently poorly understood and, while many papers discuss the modeling of neurodevelopmental disorders in hiPSCs, the literature is scarce regarding the status of XCI in hiPSCs and the potential impact on neurodevelopmental disease modeling [16–18]. In this review, we attempt to provide a concise summary of the literature regarding XCI in hiPSCs while addressing how discrepancies in the literature impact ASD hiPSC modeling.

## Review

### Induced pluripotent stem cells

Generation of hiPSCs has been performed by reprogramming of somatic cells through induced expression of four transcription factors—Oct4, Sox2, Klf4, and c-Myc. These four pluripotency transcription factors induce the pluripotent state of the reprogrammed cells by resetting transcriptional patterns as well as altering DNA methylation and histone modifications [19]. The transcription factors Oct 4, Sox2, and Klf4 are indispensable in establishing the pluripotency of hiPSCs as well as in maintaining self-renewal capacity. The transcription function of c-Myc increases the efficiency and rate of the process. Although hiPSCs exhibit many characteristics of pluripotent stem cells, they retain an epigenetic memory of the original somatic cells from which they were derived, such that they have altered DNA methylation levels and histone modifications as compared to embryonic stem cells (ESCs) [20]. A limiting factor in converting somatic cells to hiPSCs is the reprogramming efficiency. Current methods, which deliver the reprogramming activities using lentivirus, Sendai virus, or mRNA transfections, convert between 0.1 and 1.4 % of starting somatic cells into hiPSCs [21]. Additionally, it is imperative to remove the exogenous reprogramming genes to prevent the constitutive expression of reprogramming factors after the reprogramming process has been completed, as this can result in abnormal expression of endogenous genes in the reprogrammed cells as well as abnormal phenotypes, such as loss of pluripotency and differentiation [3].

### X-inactivation status in cellular models of human disease

#### Which X chromosomes are inactivated?

The current literature regarding X chromosome reactivation during reprogramming is contradictory and complex. This lack of consistency in results is apparent in research modeling Rett syndrome in human hiPSCs. For example, while results from Marchetto et al. indicate that hiPSCs undergo X chromosome reactivation (XCR), Ananiev et al. yielded results bolstering reports that the inactivation status of the somatic cell is maintained during reprogramming [22–26]. It is important to note that groups studying XCI in apparently healthy (nondisease syndrome-derived) cells have had similarly discrepant results [27, 28]. Studies in which hiPSCs have been observed to undergo XCR during reprogramming result in a cell population with biallelic activation of the X chromosomes (two active X chromosomes per cell) [22]. Alternatively, studies in which it has been reported that XCR never occurs have obtained clonal hiPSC populations exhibiting XCI [24]. These discrepant results are relevant to exploring cellular phenotypes that depend on the activation of one or both X chromosomes.

**Fig. 1:**
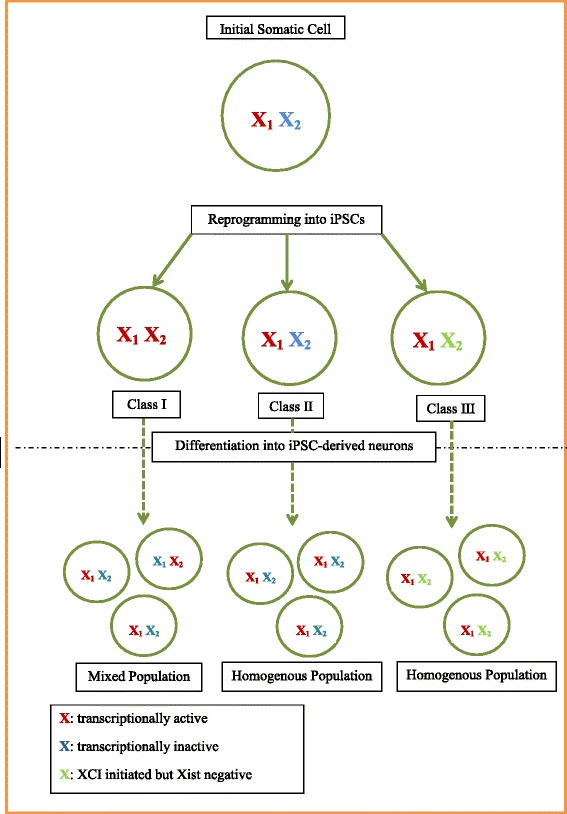
Reprogramming schematic. After reprogramming, there exist three possible combinations of X chromosome populations. In class I, both X chromosomes undergo reactivation and are transcriptionally active. Upon reprogramming, one of the X chromosomes randomly inactivates yielding a mixed population of inactivated X chromosomes. In class II, the reprogrammed population never undergoes XCR and carries over the XCI status of the original somatic cell, which is maintained through differentiation. The third outcome, class III, represents an in-between state in which the initially inactivated X chromosome loses Xist as well as H3K27me3 expression during reprogramming yet is not completely transcriptionally active. In this class, differentiation does not yield a fully inactivated X chromosome [29]. Class III is similar to erosion that occurs during extensive culturing in which an inactivated X chromosome loses Xist and H3K27me3 expression but is unable to re-inactivate upon differentiation [32]

As depicted in Fig. 1, in model systems for which hiPSCs have undergone XCR, a clonal cell population with the desired XCI event must be selected after directed differentiation (e.g., to a neural progenitor or other expandable restricted progenitor cell state), as the cells undergo random inactivation of the X chromosome during differentiation. By contrast, if the hiPSCs maintain XCI through the reprogramming process, the XCI status must be defined for each clonal hiPSC cell line and this information incorporated into the experimental design. For example, some cellular models of ASD might favor selection of a clonal population of cells with a distinct X-inactivation profile, to study the effects of an X-linked ASD mutation in both its expressed and repressed version in female neurons. However, other models that need to mimic the XCI status seen in tissues might optimally contain cell populations that are mixed or intentionally skewed to recapitulate disease mechanisms that involve skewing of XCI. A third outcome that may be obtained (also depicted in Fig. 1) represents an abnormal state of partial XCR, in which the initially inactivated X chromosome loses Xist and H3K27me3 modification upon reprogramming, yet does not fully re-express all genes of that X chromosome. Furthermore, during differentiation, this partially reactivated X chromosome does not undergo the normal process of XCI, resulting in a population of cells expressing X chromosome genes at a level above normal [29]. A table summarizing the XCI status of hiPSCs from multiple labs is summarized in Lessing et al. [29]. We reference an additional article to those cited in Lessing et al. by Barakat et al. [26].

#### Key caveat regarding mouse and human iPSCs

It is important to note that there is a fundamental difference between mouse and human iPSCs: mouse iPSCs (miPSCs) and mouse ESCs (mESCs) have routinely been observed to contain two active X chromosomes, while human iPSCs, as discussed above, have a much more variable X-inactivation/reactivation status [24]. This has profound implications for using mouse-derived cellular models of human disease that involve recapitulating the X chromosome inactivation status seen in vivo, since this property differs in mouse versus human models [24, 26].

#### Differences in XCI as a function of hiPSC type and state

The differences in XCR state among hiPSCs appear to reflect differences in the pluripotent state of the reprogrammed cells, with at least two different forms of hiPSCs resulting from reprogramming. The first human iPSC type more closely resembles murine ESCs, which are derived from the inner cell mass of the early embryo, while the second type instead has greater similarity to epiblast-derived stem cells (EpiSC). In rodent models, EpiSC are derived from the epiblast at the post-implantation stage and therefore represent a later developmental cell state than ESCs in vivo. While both types of hiPSCs are considered pluripotent, based upon several experimental criteria, ESC-like hiPSCs exhibit more naïve or “ground state” characteristics: having undergone XCR, they biallelically express the X chromosome. By contrast, EpiSC-like hiPSCs have undergone XCI and exhibit a primed rather than naïve pluripotent status [12]. These naive versus primed pluripotent cell states can also be distinguished by other criteria, such as differences in DNA methylation, altered expression levels of some pluripotency-related genes, and ability of murine ESCs to contribute to formation of chimeric blastocyst embryos, while EpiSC cannot. The molecular nature of these distinct pluripotent states, the particular culture conditions needed for maintenance or interconversion of hiPSCs with a naïve versus primed character, and the similarities/differences between rodent and human stem cell models are areas of intensive research beyond the scope of this review (for recent reviews, see [30, 31]). We focus here upon the consequences of human iPSC state upon XCI status and its relevance to using hiPSCs for cell-based models of human disease.

While the status of the X chromosome appears to differ between hiPSCs with a more naïve versus primed pluripotent character, it remains unclear to what extent hiPSCs that retain XCI versus undergoing XCR differ in their capacity for or quality of subsequent differentiation. Teratoma assays are often performed on human hiPSCs to confirm that the cells have the capacity to differentiate into multiple germ layers. Both hiPSCs that have retained XCI and those that have undergone XCR can differentiate into all germ layer derivatives in such teratomas, but further research is required to determine whether differences in differentiation capacity exist that are not appreciable by teratoma assays [22, 23].

Furthermore, the stability of XCR status in ESC-like hiPSCs is contested in the literature. In some work, XCR status is reported to be stable, enduring hiPSC line freezing and thawing as well as many passages [25]. However, other sources maintain that XCR status is transient, with spontaneous XCI occurring during culturing, resulting in a mixed population of hiPSCs with biallelic activation of the X chromosome, as well as hiPSCs that have randomly undergone XCI [26]. Several factors may contribute to the inconsistent observations of different laboratories with respect to the XCR status of hiPSCs. One possibility is that the use of different reprogramming factors or approaches may be directly affecting XCR [25]. Alternatively, it has been proposed that variation in culturing conditions of the hiPSCs may influence XCR. For example, it has been discovered that if hiPSCs are cultivated on an SNL feeder layer (mouse embryonic fibroblasts (MEFs) that express leukemia inhibitory factor (LIF)), then hiPSCs with two active X chromosomes are observed. Additionally, if LIF is added to non-SNL MEF feeder layer, XCR can be induced to some extent in previously XCI hiPSCs [27].

#### Erosion of XCI

Another possible fate for the X chromosomes of hiPSCs is erosion, which is similar to the third potential outcome of reprogramming presented above, in which the initially inactivated X chromosome loses Xist and repressive H3K27me3 modification but is not fully reactivated [29]. During extensive culturing, hiPSCs initially containing an inactive X chromosome will lose characteristics of inactivation including the XIST cloud and H3K27me3, a marker of heterochromatin. Additionally, the originally inactivated X chromosome becomes transcriptionally active [32]. Another characteristic of erosion is that XACT, a long noncoding RNA that coats the active X chromosome, spreads in eroded hiPSCs to cover the initially inactive X chromosome and precedes the loss of Xist. It should be noted that XCI erosion does not occur over the entire X chromosome but only occurs in areas of H3K27me3, a histone methylation modification associated with the transcriptionally repressed genes of the inactivated X chromosome [22, 33]. While the exact cause of XCI erosion is unknown, the consequences are very different from XCR. In eroded cells, the X chromosome is unable to undergo normal XCI during differentiation, resulting in abnormal profiles of gene expression from the X chromosome [32].

### Determining which X chromosome has undergone XCI in a clonal cell population

When studying X-linked genetic mutations, a straightforward method for determining which X chromosome is active is to assess expression of the allelic variant of the gene. This can be assayed by reverse transcription, polymerase chain reaction (PCR), and sequencing. Alternative assays may also be employed to determine the status of XCI. For example, an assay that takes advantage of androgen receptor (AR) polymorphisms utilizes methylation sensitive restriction enzymes that specifically digest unmethylated DNA; in this method, only the AR variant on the inactivated X chromosome is amplified via PCR [34]. There are two options that enable visualization of the inactivated X chromosome via microscopy that can be used to simply determine if inactivation has occurred, although these methods give no information as to which X chromosome is inactivated. One such option is to utilize RNA fluorescence in situ hybridization (FISH) to visualize Xist RNA covering the inactivated X chromosome [24]. A potential problem with this method is that Xist presence is not always a good correlate of X chromosome inactivation. As mentioned above, there are two states of X-inactivation, complete inactivation and partial inactivation, in which some transcription is occurring [35]; FISH cannot reliably distinguish between these two states. X chromosome inactivation may also be visualized by immunocytochemistry using an antibody that recognizes H3K27me3, although this may also not distinguish between partial and complete XCI.

## Conclusions

Human iPSCs represent an ethically acceptable, increasingly widely available resource for developing cellular models of disease. These models are particularly important in autism research, given the relative lack of availability of the affected primary tissue (brain) for biological studies, marked heterogeneity in the genetic influences on ASD across affected individuals (making each individual representative of a rare/unique combination of deleterious and background genetic factors), the prevalence of the condition, and the availability of effective technologies for reprogramming noninvasively derived cells from individual patients [9]. By recapitulating the unique phenotypes of specific patients, hiPSCs have the capacity to reveal cell-autonomous mechanisms influencing ASD and related disorders and to provide a platform for assessing the effects of correcting particular genetic susceptibilities.

To date, there have been few published results of hiPSC models of nonsyndromic ASD [4, 36]. Griesi-Oliveira et al. did study the effects of a mutation in MeCP2, a gene found on the X chromosome, on TRPC6, a gene believed to be associated with a nonsyndromic version of ASD. Since, in this case, the loss-of-function mutation was on the X chromosome and was severe enough to influence it to be inactivated, the status of XCR was not particularly relevant to the study [4]. While there are additional published reports involving hiPSC models of Rett syndrome, there are, again, many inconsistencies among groups regarding the status of XCR [22–24].

We conclude that variabilities in the status of X-inactivation and X-reactivation in hiPSCs are significant factors to be considered when implementing cellular models of autism and related neurodevelopmental disorders. The multiple variations in XCI status of clonal hiPSC populations include complete reactivation, complete inactivation, mixed inactivation and reactivation, and erosion. These complexities indicate that variation in reprogramming procedures and/or failure to precisely characterize XCI status can have significant consequences for the validity of a cellular model [27]. This is true for conventional technologies for reprogramming and should be clarified whenever new or emerging methodologies for transformation of somatic cells (e.g., Richner et al.) are implemented in female cellular models [37]. Careful evaluation of the extent to which X-inactivation has occurred, as well as attention to which X chromosome is inactivated in a given cell population, using available approaches involving immunocytochemistry, RNA FISH, PCR, and sequencing, are important aspects of the design and execution of hiPSC-based cellular models of disease [22, 24, 34]. It is always important to recognize that there may be other factors influencing XCI in the development of actual tissue, such as parent of origin influences, that further complicate faithfully recapitulating a human phenotype in vitro [38, 39].

The discrepancies in XCR status in human hiPSCs reviewed here underscore the need for a greater understanding of how the parameters involved in reprogramming and culturing of hiPSCs affect their pluripotent cell state, differentiation potential, and other phenotypic properties relevant to their use for modeling human disease. While it may yet take years to fully define the molecular basis of reprogramming and factors that influence acquisition and maintenance of particular pluripotent states, it is important to identify specific conditions under which hiPSCs with stable XCI status can be obtained and maintained. This will provide a reliable and standardized method for researchers to generate hiPSC models that can more faithfully recapitulate the state of the cells in vivo and that therefore have higher utility for cellular modeling of human disease states.

## Abbreviations

AR, androgen receptor; ASD, autism spectrum disorder; ESCs, embryonic stem cells; FISH, fluorescence in situ hybridization; H3K27me3, histone H3 trimethyl lysine 27; hiPSCs, human induced pluripotent stem cells; iPSCs, induced pluripotent stem cells; LIF, leukemia inhibitory factor; MEF, mouse embryonic fibroblast; mESCs, mouse embryonic stem cells; miPSCs, mouse induced pluripotent stem cells; PcG, polycomb group complex; PCR, polymerase chain reaction; XCI, X chromosome inactivation; XCR, X chromosome reactivation; Xic, X-inactivation center.
